# A Mirror-Based Active Vision System for Underwater Robots: From the Design to Active Object Tracking Application

**DOI:** 10.3389/frobt.2021.542717

**Published:** 2021-06-21

**Authors:** Noel Cortés-Pérez, Luz Abril Torres-Méndez

**Affiliations:** CINVESTAV Unidad Saltillo. Robotics and Advanced Manufacturing Group, Ramos Arizpe, Mexico

**Keywords:** underwater exploration, vision system, object tracking, robot vision, autonomous underwater vehicles, catadioptric system

## Abstract

A mirror-based active system capable of changing the view’s direction of a pre-existing fixed camera is presented. The aim of this research work is to extend the perceptual tracking capabilities of an underwater robot without altering its structure. The ability to control the view’s direction allows the robot to explore its entire surroundings without any actual displacement, which can be useful for more effective motion planning and for different navigation strategies, such as object tracking and/or obstacle evasion, which are of great importance for natural preservation in environments as complex and fragile as coral reefs. Active vision systems based on mirrors had been used mainly in terrestrial platforms to capture the motion of fast projectiles using high-speed cameras of considerable size and weight, but they had not been used on underwater platforms. In this sense, our approach incorporates a lightweight design adapted to an underwater robot using affordable and easy-access technology (*i.e.*, 3D printing). Our active system consists of two arranged mirrors, one of which remains static in front of the robot’s camera, while the orientation of the second mirror is controlled by two servomotors. Object tracking is performed by using only the pixels contained on the homography of a defined area in the active mirror. HSV color space is used to reduce lighting change effects. Since color and geometry information of the tracking object are previously known, a window filter is applied over the H-channel for color blobs detection, then, noise is filtered and the object’s centroid is estimated. If the object is lost, a Kalman filter is applied to predict its position. Finally, with this information, an image PD controller computes the servomotor articular values. We have carried out experiments in real environments, testing our active vision system in an object-tracking application where an artificial object is manually displaced on the periphery of the robot and the mirror system is automatically reconfigured to keep such object focused by the camera, having satisfactory results in real time for detecting objects of low complexity and in poor lighting conditions.

## 1 Introduction

Given the recent technological progress in the design of unmanned underwater vehicles, either remotely operated (ROVs) or autonomous (AUVs), these devices have become available to many researchers in different areas of study. In order to improve navigation of these vehicles, several methods and technologies have been used to obtain information about the marine environment, such as laser and sonar systems. Nonetheless, these technologies are in general considered invasive since both artificial-generated lightwaves and soundwaves disrupt the natural environment of marine life.

For the development of submarine vehicles or robots, vision-based systems are typically included within the set of sensors of such platforms as the main means of perception, this is due to the benefits they offer, such as the acquisition of high-resolution images, non-invasiveness and low cost.

However, in order to perform a complete autonomous navigation using only visual information, underwater vehicles need accurate and reliable information (Zelinsky, 1992; Choset et al., 2005). That is to say, the captured images must show as clear as possible, all the features of the surrounding objects in any navigation path. Thus, the obtained features must be enough to classify what is perceived in the scene, in order to detect regions of interest to be followed and obstacles to be avoided (Manley, 2003). Both cases involve computing a set of feasible trajectories in real time in order to get an autonomous-effective navigation.

Some of the challenges of underwater vision systems are strictly related with inherent conditions to submarine environments, *e.g.*, sea snow, existence of pollutants, type of local flora and fauna, changes in climate (Erdogan and Yilmaz, 2014); others are related with photometric aspects (Li et al., 1997), (*e.g.*, changes in coloration (Yamashita et al., 2007) due to the source of illumination, and eventually, those that are caused by changes in the light propagation medium. All these conditions are inevitable, and although the progress of underwater camera’s sensors technology together with the knowledge on the capturing process of objects in motion Silvatti et al. (2013) can help to reduce some of their effects, adding complexity to the system will always have an impact in the cost of developing any underwater robotic platform. In recent years, the problem of detection and tracking of underwater moving objects has received considerable attention Zuzi et al. (2018); Panda and Nanda (2020) due to wide applications in oceanographic research. It is clear thus, the need of an autonomous navigation system capable of detecting and tracking underwater moving objects of interest.

At this point, it is important to highlight two additional critical factors for underwater navigation: narrow field of vision (FOV) and image distortion. The reduction of the FOV is a crucial factor that impacts the performance of any visual navigation strategy, because a small FOV ties down information of surrounding obstacles and, therefore, limits the response capacity in maneuvers to avoid collisions. One could think that a practical solution to this problem would be to add several cameras oriented at different angles. However, distortion will persist in each camera, and what is worse, the computational cost for image analysis would increase linearly in proportion to the number of cameras.

One of the alternatives used to obtain information from the periphery, although not in a permanent way, is the use of a servo-actuated camera capable of actively varying its orientation. The use of this strategy in underwater robots also requires work for conditioning the entire device to be waterproof and to support the environment high pressures. Adding this robust system to a commercial robot entails the loss of the hydrodynamic profile of the AUV, and specially causing undesirable dynamic effects, especially for the displacement, due to the action and movement of the camera’s components and, in general, a modification of the entire design of the AUV.

Another alternative that has gained popularity among the research community in terrestrial and erial robots is the use of fisheye cameras, which offer a wide FOV, with viewing angles up to 180° (Ishibashi, 2010). However, one disadvantage that this type of lenses presents is the spatial image distortion, having more density information at the edges of the image, in other words, the information that is concentrated in the center of the image has high resolution and at the edges has very poor resolution (in terms of space pixel density). Additionally, most of these camera lenses have been designed for ground applications, where air is the interface for light diffusion. Therefore, in underwater applications (a different environment for which they were designed), the fish-eye lenses advantages are reduced (Agrawal et al., 2012).

There are research works in terrestrial environments (Okumura et al., 2011; Lee et al., 2012; Okumura et al., 2013; Lelais et al., 2019), where an active mirror system is capable of tracking high-speed particles (like a bullet) (Lee et al., 2012) using a robust and heavy recording hardware. Based on this strategy, the proposed solution has a novel mountable device based on mirror optics, which through the automatic movement of flat mirrors (Agrawal et al., 2012) can change the main angle of the preexisting fixed camera and obtain information from the periphery of the AUV.

The main contribution of this research work is to enable existing robotic platforms, which vision systems can no longer be modified, with a wider viewing angle. The proposed solution is to use active mirrors to change the angle of vision of a fixed camera system. The weight and size of an active mirror system are usually much smaller than the mechanisms used to move a complete vision system. Additional benefit is the use of existing hardware without having to invest in completely changing the design of the platform, which in most cases is impossible to perform.

Designed for ocean exploration applications, the proposed active mirror system has been experimentally tested to visually track a moving target in a coral reef environment. Based on the shape and color of the object of interest and using projective geometry, it is possible to estimate the spatial location of the object with respect to the robot’s referential frame. The visual tracking is performed using a PID control strategy to generate the motion directives of the active mirror system, having as an input reference the position of the centroid of the object in the image plane, its speed and the estimated position by using a Kalman’s filter.

To deal with the variations in color in underwater environments, we use the HSV-space color space which has been successfully reported for real-time applications due to its low computational cost. To deal with possible sunlight reflections, the system’s own design conceals the mirror system of direct Sun exposure with the robot’s own body. For the analysis of active mirror visual information, a bi-cubic interpolation method is used to compute the projection of points that belong to mirror area, then a homography is computed to extract an image blob of the reflective surface.

The outline of the paper is as follows. Section 2 presents detailed information about prototype design, the kinematics of the mirror movement as well as a virtual camera model. The process of image analysis (segmentation and extraction of the mirror projection region) is contained in Section 3, as well as the method used for object tracking using a segmentation process in the HSV color space. The control algorithm of the mirror system is presented in Section 3.5, where all the blobs are geometrically weighted to estimate the centroid of the object, then a Kalman Filter is performed to predict the object velocity in image in order to implement PID control. The experimental results and analysis of the performance are presented in Section 4. Finally, the conclusions and future work are given in Section 5.

## 2 Mirror System Configuration and Design

Several research work on robot navigation and exploration have successfully tested active-camera vision systems (Manley, 2003; Yahya and Arshad, 2016; Vidal et al., 2018). Despite the diversity of approaches in these works, the pan-tilt movement scheme has been widely chosen because of its simplicity. Our work continues on this trend but instead of using a mechanism to directly generate directly pan-tilt motion in the camera, we use mirrors.

The idea of using mirrors in the design of our active system is inspired by the mechanism of the single-lens reflex camera (SLR). In this type of camera, a moveable mirror behind the lens reflects an image through a pair of mirrors, onto the viewfinder. Thus, in an inverse approach, one could think of changing the direction of light beams from around the robot and focus them to the camera sensor with the appropriate motion of the mirror. Taking this approach as a reference, it has to be considered the reflection of the principal axis of view and that the mirrors must be on the camera’s FOV.

**FIGURE 1:**
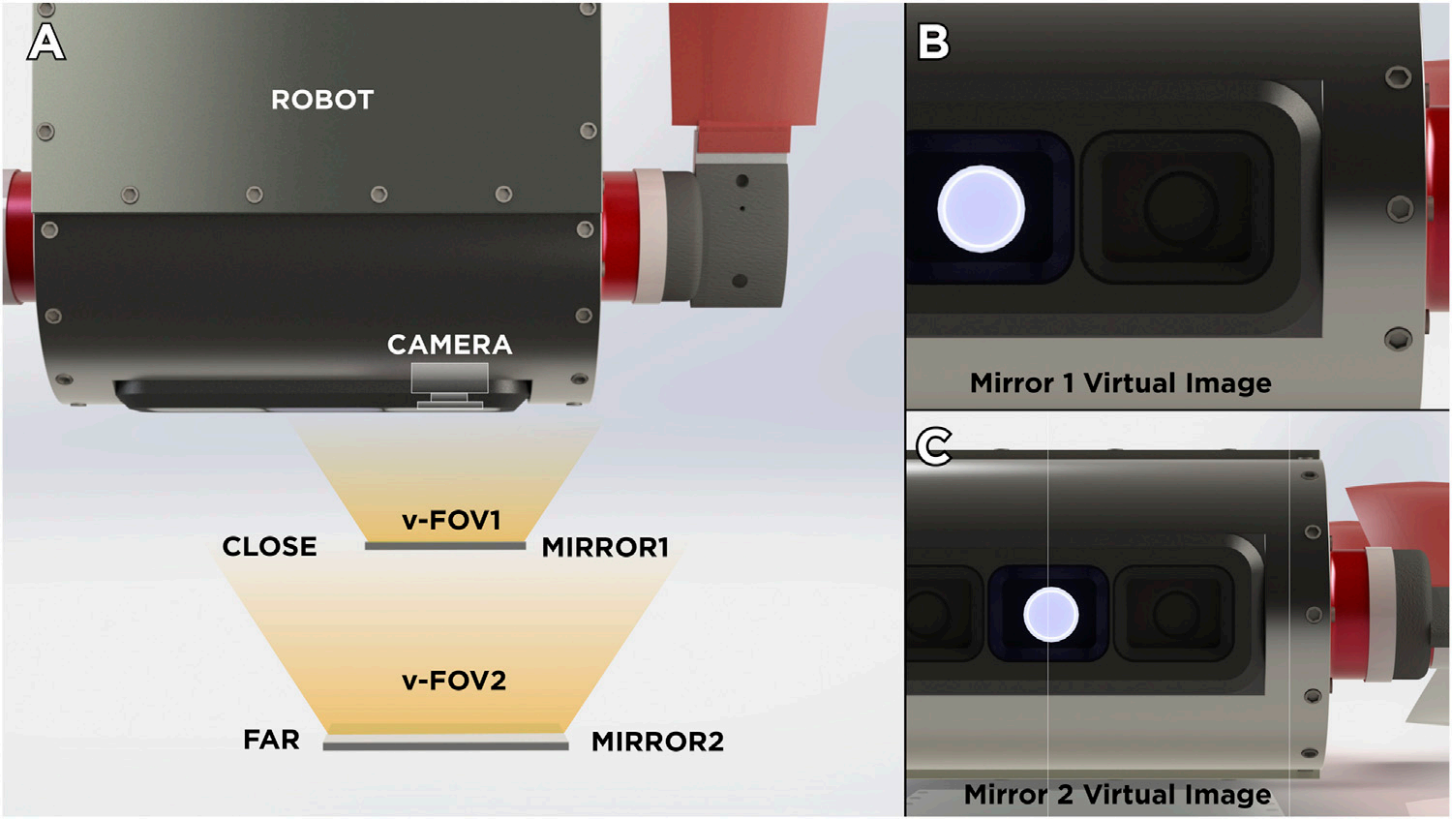
Illustrative comparison of virtual images produced by the mirror position. **(A)** General Overview of two scenarios. **(B)** Virtual Image produced when mirror is placed near from the robot’s camera. **(C)** Virtual Image produced when mirror is placed near from the robot’s camera.

In this section, the details on the design and the kinematics of the mirror system is described. First, we address a fundamental aspect in the design of any mirror system: the distance between the mirror and the camera. On one hand, if the mirror is placed very close to the camera, the virtual image on the mirror could present occlusions caused by the reflection of the body of the robot, thus dramatically reducing the useful field of view of the device (see Figures 1A,B). On the other hand, if the mirror is placed too far from the camera (Figure 1C), it would require a very large mirror in order to use the majority of the image area due to the perspective. In addition, for applications involving underwater or even erial vehicles, if we opt for a long support with a mass at the end (mirror), it could change dramatically the center of mass of the system which in turn will change the dynamic parameters needed for dynamic-based controllers. In Okumura et al. (2011), the authors try to solve this problem by using a set of lenses called “pupil transfer lenses” as an alternative to reduce mirror size. However, for the proposed development, this is not a feasible solution for underwater environments, due to variations in light propagation.

**FIGURE 2:**
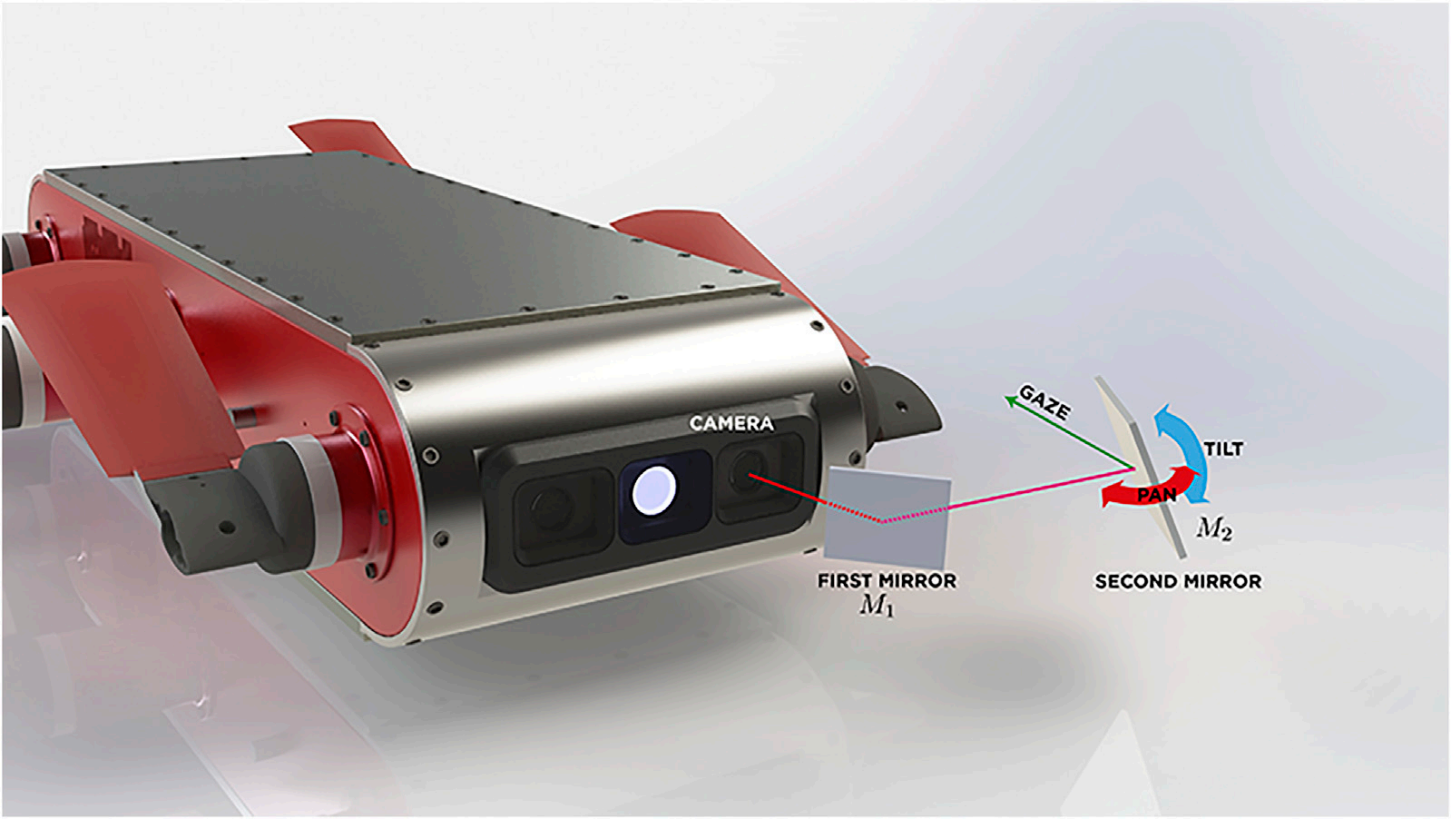
A two-mirror system design interacting with the AUV’s camera.

To address possible robot-body occlusion a system composed by two mirrors $M_{1}$ and $M_{2}$ is proposed. Similarly to a pentaprism in an SLR camera, two mirrors change the direction of view for the camera user. Using this approach, the target of $M_{1}$ is to turn aside the camera’s view direction toward $M_{2}$, thereby $M_{2}$ is responsible for changing the gaze direction by moving in two axes and generating a virtual camera. The virtual camera corrects image inversion caused by $M_{1}$, targeting principal view axis toward the desired direction of observation. Figure 2 shows this interaction between the two-mirror system and the AUV’s camera.

### 2.1 Virtual Camera Kinematics

One of the disadvantages of including a system of two mirrors in relation to the system of a single mirror is that the mathematical model of motion of the virtual camera grows in complexity. However, with some design considerations, it is possible to simplify the motion model of the system.

The first aspect that can been taken into account to simplify the kinematic model is to consider a constant translation between the reference frames of each of the mirrors, then the pose of the first mirror $M_{1}$, with referential frame ${\text{Σ}}_{1}$, can be considered constant over time. The gazing mirror $M_{2}$ changes its pose and consequently the pose of the virtual camera being driven by two motors, which will be represented by the generalized coordinates $q={\left[q_{1},q_{2}\right]}^{T}$, where $q_{1}$ and $q_{2}$ are the tilt and pan angles, respectively. The model complexity is reduced given that the axes of rotation intersect at the center of the mirror surface and the angle of the first mirror is fixed.

**FIGURE 3:**
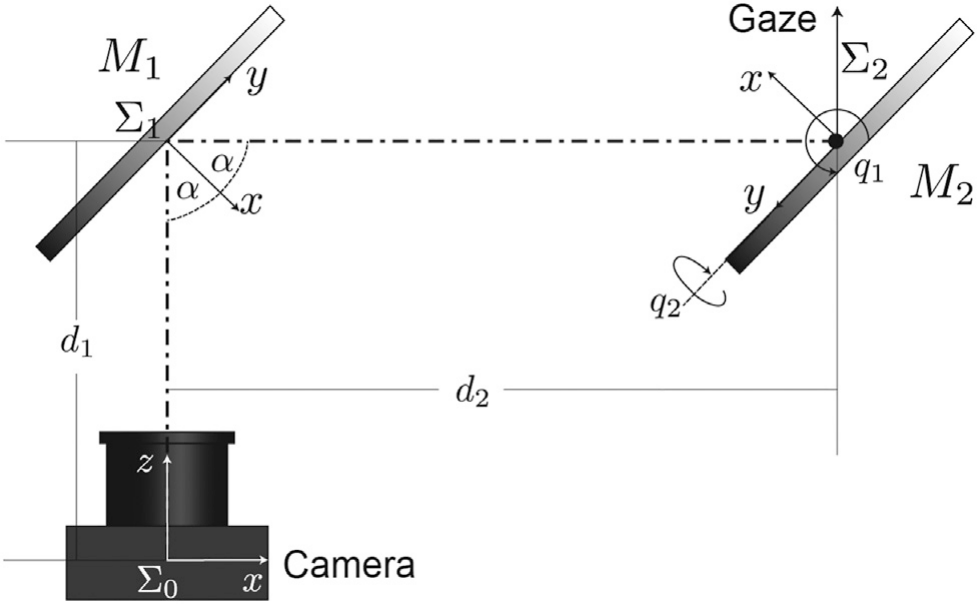
Reference frames of the system. Since we assume a system of fixed cameras on the robot, we consider a frame $\Sigma_{0}$ at the origin of the camera. The origins of the frames $\Sigma_{1}$ and $\Sigma_{2}$ are located in the center of the mirrors *1* and *2*, respectively.

A simple scheme of this mirror-camera arrangement is represented in Figure 3. It is assumed that reference frame ${\text{Σ}}_{1}$ is attached to $M_{1}$ and ${\text{Σ}}_{2}$ is attached to $M_{2}$, which has a pan-tilt movement and is also displaced a distance $d=d_{1}+d_{2}$ (see Figure 3) w.r.t. the base frame ${\text{Σ}}_{c}$, which in this case yields to reference frame assigned to camera.

First transformation (rotation and displacement $d_{1}$) between both reference frames ${\text{Σ}}_{0}$ and ${\text{Σ}}_{1}$, is a constant transformation given by:

$$T_{0}^{1}=\left[\begin{array}{cc} \left[R_{\lambda_{\left(\alpha \right)}}\right] & d_{1}\\ 0 & 1 \end{array}\right]\in SO_{\left(4\right)},$$ (1)

where $R_{\lambda_{\left(\alpha \right)}}$ is a rotation matrix over the λ axis, where $\lambda \in {\mathbb{R}}^{3},\lambda =1$, *α* is the value angle of rotation which is always $\alpha =\frac{\pi }{2}$. Notice that since $d_{1}$ is only displaced in the direction of the positive z−axis of the camera, we have $d_{1}={\left[0,0,|d_{1}|\right]}^{T}$. The matrix that transforms the values expressed in the ${\text{Σ}}_{2}$ reference frame to the ${\text{Σ}}_{1}$ reference frame is given by:

$$T_{1}^{2}=\left[\begin{array}{cc} \left[R_{z_{\left(q_{1}-\frac{\pi }{2}\right)}}R_{y_{\left(q_{2}-\frac{\pi }{2}\right)}}\right] & d_{2}\\ 0 & 1 \end{array}\right]\in SO_{\left(4\right)}.$$ (2)

As mentioned above, we consider the origin of the reference ${\text{Σ}}_{2}$ in the center of the second mirror. Thus, we have the center of the mirror located in ${\;}^{2}X_{e}=\left[0,0,0,1\right]$. Please note that the upper index 2 indicates the reference frame. Note also that by placing the reference frame in the center of the mirrors and the center of rotation axis, this vector is invariant for all values of generalized coordinates vector *q*. Therefore, we have the vector $X_{e}$ expressed in the camera frame as:

$$X_{e}=T_{0}^{1}\;T_{1}^{2}{\;}^{2}X_{e}.$$ (3)

**FIGURE 4:**
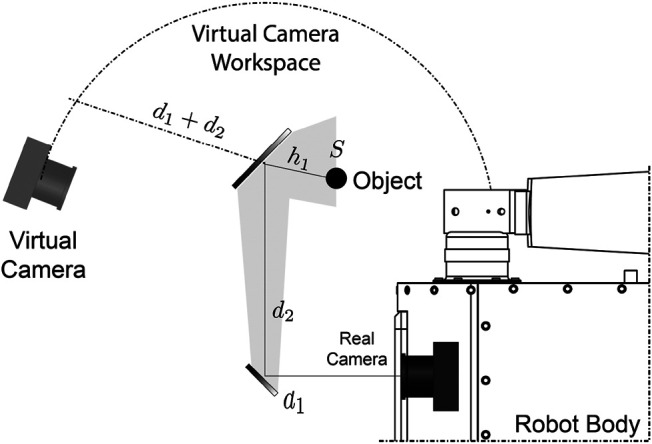
Virtual camera workspace. Since $d_{1}+d_{2}$ remains constant, the locus where the camera moves is an arc of radius $r=d_{1}+d_{2}$ for 2D projections, and the surface of an sphere for a 3D space with the same radius.

So far, we have only discussed about the description of the movement of the center of the mirror with respect to the center of the camera as a function of the joint coordinates $q_{1}$ and $q_{2}$. However, what is in our interest is the description of the movement or pose of the virtual camera with respect to those joints. One of the optical phenomena of mirrors is the formation of a virtual image with depth equal to the actual distance of the object to the mirror (see Figure 4). This property could be used to describe the center of the virtual camera. As shown in Figure 4, the position of *S* (representing a virtual object produced on the mirror) keeps the same displacement $h_{1}$ in the direction of the specular surface. However, the displacement $h_{1}$ on the normal axis to the mirror surface is negative. Thus, we have that the movement of the objects reflected on the mirror are symmetrical on the plane of the mirror. In a reverse approach, we can say that the movement of the virtual camera is symmetrical in the plane of the mirror relative to a static light source.

As it can be seen, the mapping of the camera’s motion is nonlinear, thereby it is difficult to model an inverse relationship (*i.e.*, there is not a unique inverse mapping) that allows us to choose a joint configuration given a desired position of the virtual camera. However, the true objective and challenge in most of the active vision systems, is to find the necessary variations for a system reconfiguration.

Different numerical and artificial intelligence methods have been tested in order to solve this inverse mapping, restricted to joint boundary and initial conditions. In this research work, we focus on the development of the active vision system, its kinematics model and its use in a real environment applications. However, at this point, with a general model it is not possible to describe completely motion constraints as well as the practical functional limits (considering mirror pose). These constraints depend entirely on the specific design of specific prototype dimensions as well as the AUV to be used. However, by ensuring these design landmarks, a different prototype can be adjusted for a specific AUV platform.

### 2.2 Prototype Design for the AQUA 2.0 Robot

We have considered so far a double mirror system as a generalized way to study kinematics, however, factors such as the AUV platform, camera parameters, etc., must always be considered for the design of any prototype.

To follow the design line we present, we need to analyze first the physical configuration of our AUV’s camera, *i.e.*, to which direction it is pointing at and its FOV. In this case, and without losing generality, the AUV robot platform considered for our prototype of the active mirror system is an AQUA 2.0 robot manufactured by Independent Robotics (Georgiades et al., 2004). This amphibious robot has a two-camera system on the front in a stereo configuration, which does not allow visual information on the sides and rear of the robot (an additional camera is located on the back and it is commonly used to give commands via QR code tags). Although this AUV is thrusted by fins and not by propellers, this platform reflects many similarities with other systems which also do not have an active vision system. From this point forward, mirror system design discussed in this article is based on the physical properties of this robot.

The second aspect to consider, being one of the main discussions about the prototype design, is the position of the first mirror $M_{1}$ with respect to the body of the robot. On the one hand, we want to reduce the impact on AUV dynamics when adding external objects but, on the other, we also want to reduce possible occlusions and maximize the useful virtual camera workspace by reducing the possibility of obstructions in its view. For this reason, we decided to place the observation mirror under the body of the robot, so it is necessary for the first mirror to direct the axis of view downwards with an inclination of 45° from the vertical, thus producing a deviation of 90° according to Snell’s law (which describes the light reflection on mirror surfaces). This configuration also allows both mirrors to be covered from direct exposure to the Sun’s rays, as the downward-facing mirror is fully covered and the same body of the AUV covers the observation (gazing) mirror in most cases.

Now, with the main optical axis of the camera deviated downwards, the third aspect to consider in the design is that our current vertical view axis must coincide with articular axes of the gazing mirror (which are coplanar), to simplify the kinematics and, consequently, the controllability of the overall system.

**FIGURE 5:**
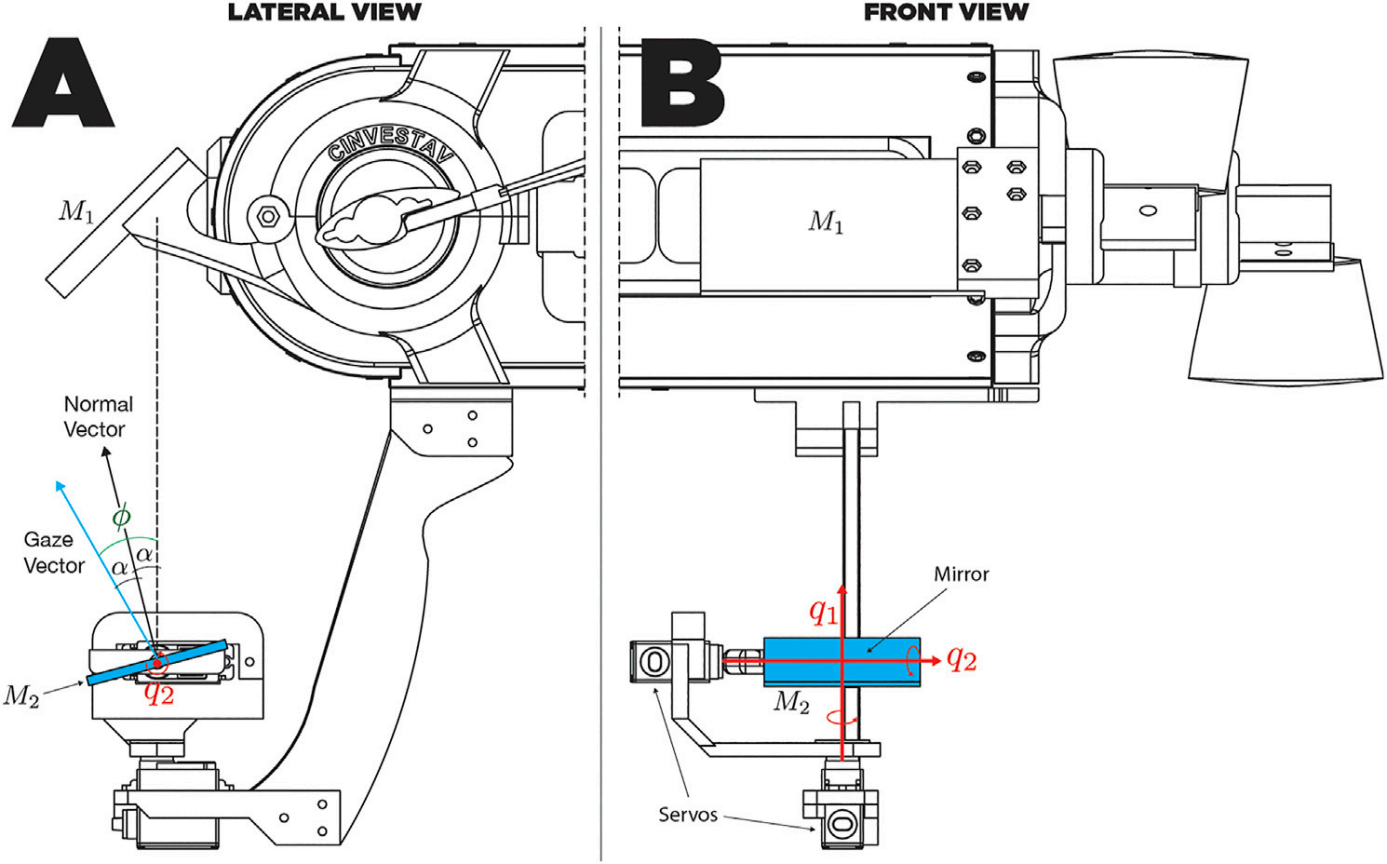
Lateral **(A)** and frontal **(B)** view schemes of the mirror system.

**FIGURE 6:**
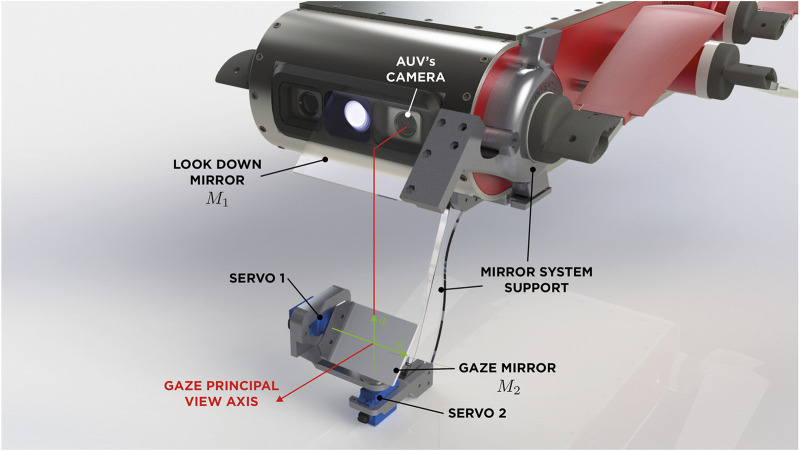
Schematic diagram of the structure and degrees of freedom (DOFs) of the gazing mirror. $q_{1}$ moves the mirror in *roll* and $q_{2}$ in *pitch*.

Taking into account these three simple aspects, we can design a mirror system according to the platform we are working with. Figure 5 shows an overview of the full vision system mounted on the AUV, showing lateral (Figure 5A) and frontal (Figure 5B) views. It can be noted in the front view (Figure 5B) that the variation of $q_{1}$ will cause a rotation motion over a vertical axis, which in turn will cause a rotation in the angle of vision. This rotation will be in the same ratio as the movement of $q_{1}$ since the projection of the main optical axis always affects with the same angle on the plane of the mirror, independently of the value of the joint. Figure 6 shows a scheme of the prototype’s design for the specific platform AQUA 2.0, where mirrors are interacting with the robot’s camera. Since the looking-downward mirror remains always in the same position with respect to the camera frame, the gazing mirror has full control of the system’s view direction through its orientation.

### 2.3 Spherical Vision Range, Analysis and Considerations

By varying the pose of $M_{2}$ rotating over $q_{2}$, we can directly control the direction of the normal to the surface, which is our main interest for our control issue since we can also deduce that the control on the pitch axis of the virtual camera and the resulting line of sight is given by the angle *ϕ*, measured from the main optical axis. Thus, it is also easy to see that ϕ=2α, which is a relationship commonly used to describe the angle of reflection in a specular surface.

For this case, what really matters is the opposite relation, because the reflection vector in this analysis denotes the gaze vector of the mirror system. To do this, we will define the state of the system (orientation of the virtual camera) as:

$$V_{cam}=\left(\begin{array}{c} \theta \\ \phi \end{array}\right).$$ (4)

There is a direct dependency between the value of *θ* that describes the azimuthal value of the virtual camera with the value of $q_{1}$. The relationship between *ϕ* with respect to $q_{2}$ has already been described and then it is easy to see that the inverse relationship is given by:

$$V_{cam}=\left(\begin{array}{c} \theta \\ \phi \end{array}\right)=\left(\begin{array}{c} q_{1}+\theta_{offset}\\ 2\left(q_{2}+q_{2offset}\right)+\phi_{offset} \end{array}\right),$$ (5)

where $\theta_{offset}$ is an offset value to align the references between the control value of $q_{1}$ and *θ*, $q_{2offset}$ is a constant offset value to align the references between the control value $q_{2}$ and the main optical axis vector. Finally, $\phi_{offset}$ is an offset value to align the references between $q_{2}$ and *ϕ*.

For rotational joint systems there are no unique inverse kinematics solution and this case is not an exception. In this particular case, the different solutions (set of joint values) can achieve the desired orientation of the virtual camera in the same period ($0^{\circ }-360^{\circ }$) –although this is purely theoretical since most servomotors have joint limits with a range of $180^{\circ }$.

Physical movement constraint of actuators is not a trivial fact, because as it has been expressed in Eq. 5, the variable *θ* is only dependent on $q_{1}$, as well as *ϕ* is only dependent of $q_{2}$, which limits the range of motion of the virtual camera to the range of motion of the actuators. However, by analyzing the relationship between *ϕ* and $q_{2}$, we realize that the variation of $q_{2}$ originates a variation of double the movement in *ϕ*, so a range of movement of $180^{\circ }$ in $q_{2}$ is enough to cover a range of $360^{\circ }$ in *ϕ*, theoretically. However, this is not possible because virtual images are not produced for configurations where the specular surface is out of the camera’s FOV. Integrating all movement range of $q_{1}$ and $q_{2}$, we observe that we can theoretically vary the direction of the resulting axis of vision in a workspace similar to a half of sphere.

**FIGURE 7:**
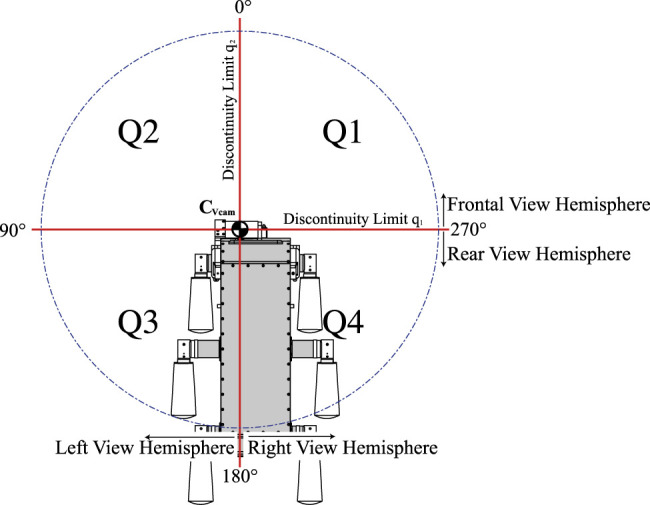
Spherical vision range of the mirror system divided in quadrants.

Figure 7 assumes an elevation of $0^{\circ }$ in the looking direction of the virtual camera and divides the regions in the periphery into four quadrants. The challenge in this configuration will be given only by the discontinuity that the every spherical coordinate system presents in zenith, being limited by the speed and control strategies to avoid these singularities.

## 3 Methods

In the previous section, we established a mirror system configuration given a specific platform. We now present the general method for tracking a moving object, which first extracts the visual information provided by the virtual camera and then performs an overall control system to be used for navigation.

### 3.1 Image Extraction

**FIGURE 8:**
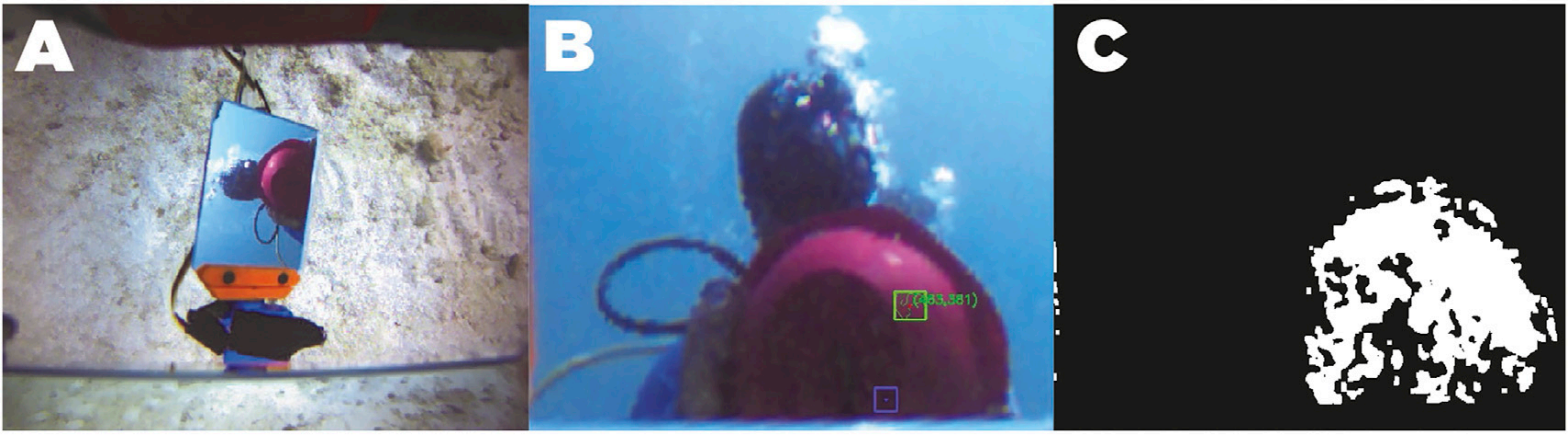
Red ball detection using HSV color space linear classification. **(A)**: image acquired in the RGB space. **(B)**: Homography projection of the active mirror region. **(C)**: Color threshold map in the HSV space.

Due to our AUV’s load capacity, dynamics alteration, camera perspective and other limitations already mentioned, the chosen size of the mirrors may not cover the entire FOV. A guide on how to determine the size is presented in Cortés-Pérez and Torres-Méndez (2016). This fact will cause a scene divided into three main regions, which corresponds to different directions in the vision traces: the regular view region, the passive-mirror view region ($M_{1}$ projection) and the gazing-mirror view region ($M_{2}$ projection). Figure 8 shows a typical capture of the AUV’s camera through these three main regions. Each region in the image has different characteristics that could be exploited by different navigation approaches. Figure 8A shows the image acquired in the RGB space. Of course, the region of interest in our research is the region that corresponds to the active mirror. Since prototype mirrors are rectangular-shaped, its projection on the image will ideally be a quadrilateral, which can be roughly delimited by the projections of its corners in the image plane. To perform navigation tasks, this not so sophisticated approach seems to be ideal due to the low computational cost compared to more precise segmentation methods, so the problem of extracting visual information from the gazing mirror is to calculate the projections of such coordinates given an articular configuration $q={\left[q_{1},q_{2}\right]}^{T}$. There are two possible ways to do it:

1. **Projective geometry**: Analytical methods for computing projections by knowing camera calibration parameters.
2. **Numerical Methods**: These methods included a wide variety of analytical, heuristic, probabilistic, and machine learning methods. Require a training phase for tuning the model numerical parameters.

We decided to use a numerical method, but before describing it, we present the reasons for not using an analytical method.

#### 3.1.1 Analytical Methods for Computing Projections

These methods have been extensively studied for 3D vision applications, for both inertial and mobile robotics vision systems, however, calibration of camera parameters in underwater environments is complicated as the light propagation medium changes the geometrical and photometric properties of vision systems (Manley, 2003; Yamashita et al., 2007). Yamashita et al. (2007) describe some of the phenomena of light interaction with the camera along with the propagation properties in the aqueous medium and through the camera lens.

To exemplify how the light direction varies with respect to the camera sensor, several camera calibration tests were performed in our laboratory, using GoPro Hero 4 and Samsung gear 360 2017 (with fisheye lenses) cameras equipped with underwater housings. Both cameras where calibrated using MATLAB camera calibration Toolbox at three different media: air, distilled water and salt water.

**FIGURE 9:**
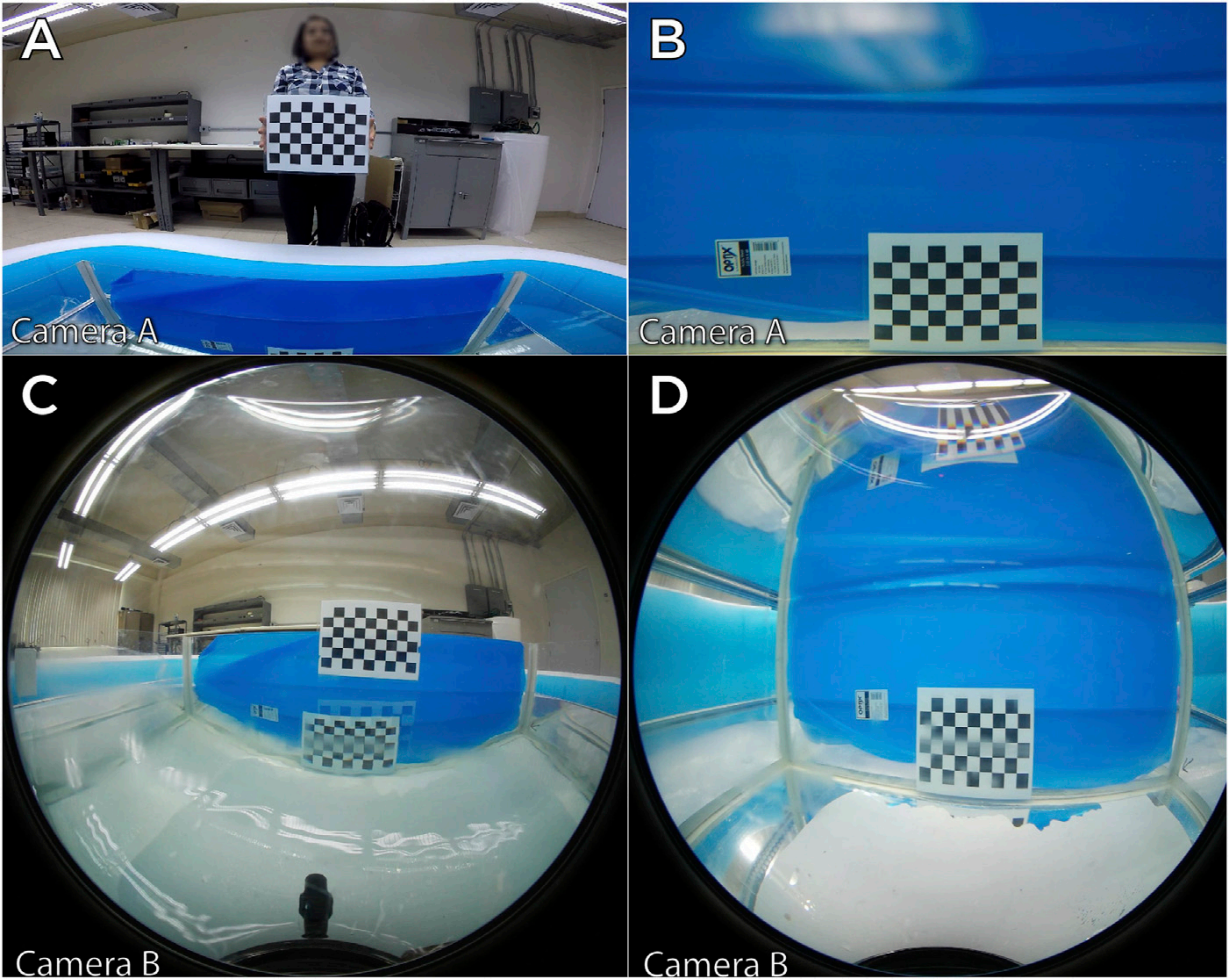
Comparison of two wide angle and fisheye cameras in different propagation medium. **(A)**: wide angle camera on air. **(B)**: wide angle underwater. **(C)**: fisheye camera on air. **(D)**: fisheye camera underwater.

In Figure 9, we can observe that in both cases the dimensions of the calibration board change dramatically when entering an aqueous medium, it can also be appreciated that the FOV is reduced.

To quantify this alteration, we can review the result of the camera calibration process and compare how parameters vary (see Table 1) in the different media with a concentration of 30 parts per thousand. Note that the focal parameters are noticeably modified as well as the center axis (standing for different radial distortion).

**TABLE 1:** Camera calibration parameters in different environments.

Cam 1	$f_{x}$	$f_{y}$	$c_{x}$	$c_{y}$
Air	476.42	475.98	960.68	744.18
Distilled water	876.80	870.91	927.34	756.74
Saltwater	879.63	871.17	926.43	758.05

Regarding the extraction of the corners of our mirror, in order to effectively use an analytical projection method, it is necessary to have an accurate calibration of the actuators, which adds another challenge for the system.

#### 3.1.2 The Nearest Neighbor Method

For the mentioned reasons in the previous section, we use a numerical method to find the corners that define the projection of the active mirror. The method we use for the segmentation is known as “*the nearest neighbor.*” This method fits in the second category, particularly, in the classification of interpolation statistical methods. The algorithm of the nearest neighbor selects the value of the nearest point and does not consider the values of all the neighboring points of the whole, producing a precise and constant interpolation. The algorithm is simple and is commonly used for 3D rendering.

In this method, *N* samples of different heights need to be considered, that is, we have *N* triads $\left(q_{1i},q_{2i},z_{i}\right)$, where $z_{i}=f\left(x_{i},y_{i}\right)$. In this process, the estimation of $z_{i}$ is desired given a set of articular values $q_{1}$ and $q_{2}$. To estimate $z_{i}$ we have:

$$z=\{\begin{array}{c} \frac{{\text{Σ}}_{i=0}^{N-1}\frac{z_{i}}{{\left[{\left(x_{i}-x\right)}^{2}+{\left(y_{i}-y\right)}^{2}\right]}^{p/2}}}{{\text{Σ}}_{i=0}^{N-1}\frac{1}{{\left[{\left(x_{i}-x\right)}^{2}+{\left(y_{i}-y\right)}^{2}\right]}^{p/2}}}\\ z_{i}\;\text{other case}, \end{array}\;\;\forall x_{i}\neq x\;or\;y_{i}\neq y,$$ (6)

where p generally determines the relative importance of distant samples. Note that the denominator gives a measure of how close the estimated point of the samples is. Naturally, if a sample is close, then it has a greater influence on the estimate.

In the case of estimating the corners in the active mirror, we have the configuration of the servomotors $q_{1}$ and $q_{2}$ as input of the model, that is to say $x_{i}=q_{1\left(i\right)}$ and $y_{i}=q_{2\left(i\right)}$. Then, we have a function $f_{j}$ for estimating each coordinate of the set of points P(x,y), that is: eight interpolation functions (model outputs), which represent the four corners of the active mirror (composed by the two coordinates *x* and *y*).

To accurately calculate each of the functions, it is necessary to have sufficient and representative information in the training data set covering the entire workspace of the mirror system. Unfortunately, this is impossible to perform it online. However, once the parameters for the regression have been calculated, the interpolation method turns out to be very fast, making it suitable for implementation in real-time applications.

Finally, to extract the visual information enclosed in the quadrant, a homogenous transformation of each of the pixels contained within the convex polygon of interest was performed to obtain an image of constant dimension that represents the rectangular surface of the mirror. In this way, any analysis will be based only on the visual information of the active mirror. Figure 8B shows an example of the projection of the pixels contained in the active mirror by means of the calculated homography matrix.

### 3.2 Target Detection

In 2015, a contest was launched in Piombino, Italy, for the detection of buoys with underwater robots. Balazs Suto *et al.* developed a method for the detection of yellow buoys in this contest based on the change of color space (Sütő et al., 2015). It should be noted that in this test the location of buoys was made in the open sea, always having a contrasting color between the blue background and the buoy. However, since our work is oriented toward the exploration of coral reefs, which are environments rich in colors, we decided to have a red ball as our artificial object to be tracked, being red the first frequency that is lost (Sütő et al., 2015), this represented an additional challenge.

There are several algorithms based on pixels, gradients, textures and many other descriptors for object detection in underwater vision systems (Sudhakar and Meena, 2019). Some of the most used methods for circle detection are based on the Hough transform, which require a high computational cost, which translates in a slow performance for a system with limited computational capabilities, as is the case with most the AUVs. Other methods, such as EDCircles, use edge information incorporating edge detectors such as prewire, Sobel and Canny and, although they have been successfully tested for underwater images, they depend on good scene lighting for the effective computation of gradients, which it is not guaranteed at great depths (**?**).

Given that the object to be tracked is of red color and of circular shape, the key part of the detector is to identify the color of the pixels that belong to the ball. However, due to the photometric properties and light interactions underwater, the “red” color will not be uniform, thus the color detector must be robust to all the range of gradients according to shading. In addition, as the color of the ball will be affected to depth (in the sea), the detector had to be also robust to saturation. Although we know that the target object has a specific geometry, we also know that edge-detection based methods, which use frequency information for compute gradient descriptors (like Canny and Sobel), will have a poor performance compared when they are used in a well lit enviroment (Sudhakar and Meena, 2019). We decided to use the HSV color space similar to the work of Balazs Suto *et al*, which has proven to have robustness properties for color classification in addition to having a lower computational cost.

Notice that by selecting an appropriate saturation window (*S* channel), a linear classification with the thresholds of the “red” tones in the hue channel could be made, which would be a complex function if the representation is made in the RGB color space. Therefore, only the homography projection image is processed to detect the red ball, where the color in the HSV space is trimmed with hue values corresponding to the red tone. Figure 8C shows the resulting color threshold map in the HSV space. Then, we use circular-shaped morphological filters of erosion and dilation to reduce possible holes in the binary segmentation mask, thus detecting the least number of red spots in the image. OpenCV tools are used in the image in order to find contours which will delimit color clusters in the image. Then, all possible clusters that could contain a circle are searched, determining its area and its center. The controller will use the information of the centroid of these spots to actively keep the view in it, moving the actuators of the active mirror.

### 3.3 Colored Spot Clustering

As a measure to reduce the noise of multiple pixel clusters detected, in the control strategy each of the colored spots received from the detection stage are weighted by using its area and location of its centroid. To do this, we made a geometric average considering giving more weight to the spots of greater area for the calculation of a single centroid. The equation that describes this weighting is:

$$C_{x,y}=P\left({\text{Σ}}_{i=1}^{N}\frac{\lambda_{i}x_{i}}{A},{\text{Σ}}_{i=1}^{N}\frac{\lambda_{i}y_{i}}{A}\right),$$ (7)

where $\lambda_{i}$ is the area of each colored spot, $x_{i}$ and $y_{i}$ are the coordinates of the centroids and *A* is the sum of the areas of all the spots, that is $A={\text{Σ}}_{i=1}^{N}\lambda_{i}$.

**FIGURE 10:**
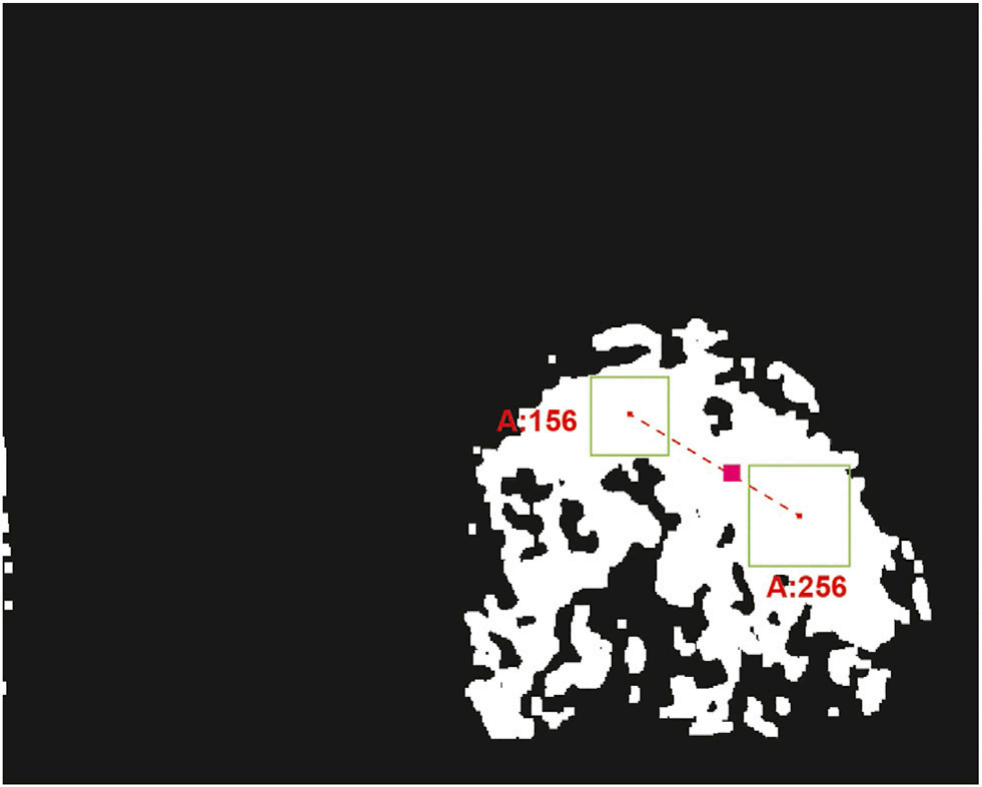
Centroid estimation of the ball by pondering all detected clusters. This figure shows a mask where the black pixels represent visual information completely outside the range of colors of the red ball, defined in the HSV color space. The white pixels represent the part of the image that has color information within the established range. The green boxes represent two spots detected. The pink line joins green boxes centroids and the pink box is the estimation of the centroid given to the position and areas of green boxes.

Figure 10 shows an example of the centroid estimation when the detector finds two colored spots. In this case, the first spot has an area of 156 pixels and the area in the second spot is 256 pixels. The estimated centroid of the object is shown in the figure with a pink square. It can be seen that the centroid is on the line that joins the centroids of the spots, closer to the right-down region, which is where the stain has the largest area.

### 3.4 Kalman Filter

In order to reduce this noise, we incorporate a Kalman filter to estimate the state from the previous measurements, helping to generate references of the object of interest when it leaves the field of vision of the active system. Once the possible centroid of the object has been evaluated this information is used for location of this point on the homography projection image. The Kalman Filter equations are as follows:

$$x_{k}=A_{k-1}x_{k-1}+B_{k-1}u_{k-1}+w_{k-1},$$ (8)

$$z_{k}=H_{k}x_{k}+v_{k},$$ (9)

where the state of the system at the *k*-th time instant $x_{k}$ is represented by:

$$x_{k}=\left(\begin{array}{c} Cx_{k}\\ Cy_{k} \end{array}\right),$$ (10)

which are the centroid coordinates at time *k*. Also, in Eq. 9, we have that $w_{k}$ is the corresponding white noise with an average value equal to zero and with variance $Q_{k}$. $v_{k}$ is white noise with an average value equal to zero and with variance $R_{k}$ at time *k*. $A_{k-1}$ is the state matrix and $B_{k-1}$ is the input matrix, both at the instant k−1. $H_{k}$ is the output matrix at the *k* instant.

### 3.5 Gaze Control

Since the estimation of the object’s centroid is in the image plane, our visual control works by keeping the centroid of the object of interest always on the main optical axis, that is, at the center of the homography transformation. The system error $e_{x,y}$ then is defined as:

$$e_{x,y}=\left[C_{x}-\frac{1}{2}I_{w},C_{y}-\frac{1}{2}I_{h}\right],$$ (11)

where $I_{w}$ and $I_{h}$ are the width and height of the homography image, respectively. We also define an error vector of the prediction $e_{p\left(x,y\right)}$ as:

$$e_{px,y}=\left[p_{x}-\frac{1}{2}I_{w},p_{y}-\frac{1}{2}I_{h}\right].$$ (12)

Note also that $e_{p}$ is directly related to the error in the pan direction of the system and $e_{x}$ with the tilt error. Finally, the control law used for the object tracking with the mirror system is:

$$q_{1\left(k\right)}^{d}=q_{1\left(k-1\right)}+kp_{1}\left(e_{x\left(k\right)}+\psi e_{p\left(x\right)\left(k\right)}\right),$$ (13)

$$q_{2\left(k\right)}^{d}=q_{2\left(k-1\right)}+\frac{1}{2}kp_{2}\left(e_{x\left(k-1\right)}+\psi e_{p\left(x\right)\left(k-1\right)}\right),$$ (14)

where $kp_{1}$ and $kp_{2}$ are the movement gains of the actuators $q_{1}$ and $q_{2}$, respectively and *ψ* is an enabler defined as:

$$\psi =\{\begin{array}{cc} 1 & \text{ if object is found}.\\ 0 & \text{if object is not found}. \end{array}$$ (15)

## 4 Results

Field experiments were conducted by deploying our underwater platform at 10–12 m of depth in a coral reef environment located in Costa Maya, Mexico. We used the left front camera of the robot to mount our prototype mirror system built of ABS plastic parts printed on a 3D printer (substantially decreasing the cost) to which we add a finishing surface based on automotive paint and ceramic lacquer coat for hardening.

**FIGURE 11:**
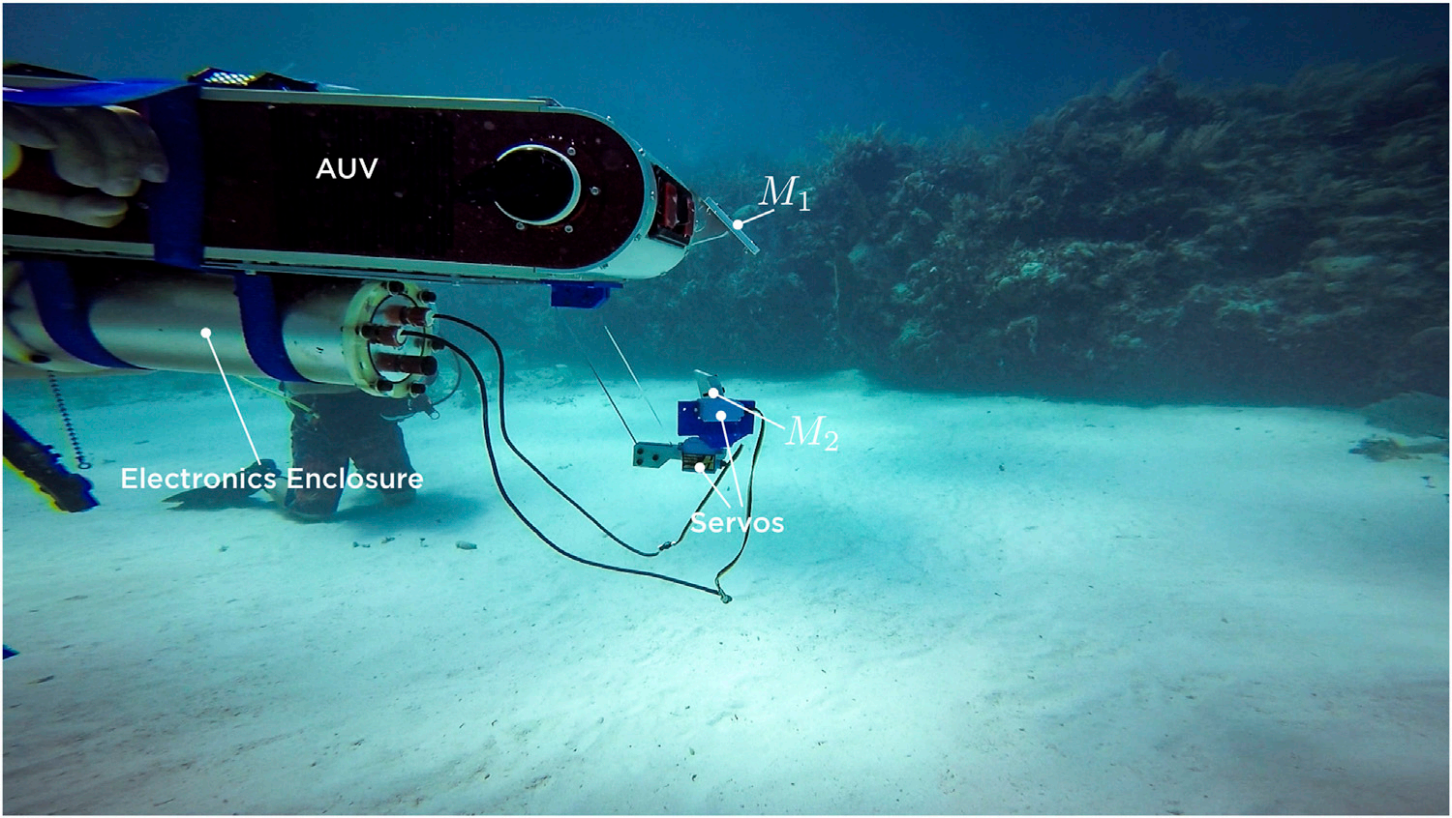
Photography of the prototype of mirrors mounted in the AUV in the field trials at 10 m deep. The cylindrical module under the robot contains all the control electronics of the mirror system, as well as the communication circuits by means of optical fiber with the robot’s computer.

**FIGURE 12:**
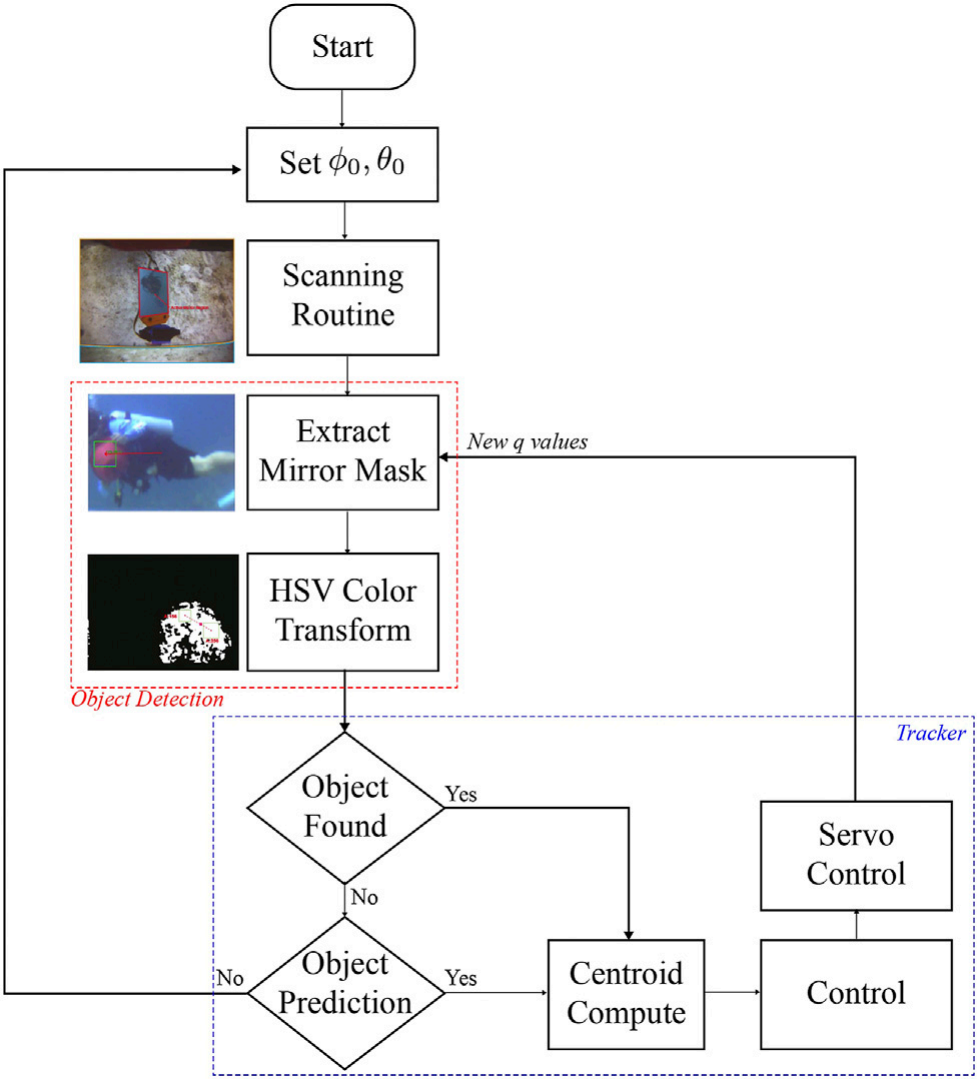
Flowchart of the active tracker for experimental trials. The red line frames the process of detecting an object from colored spots in the HSV space. The blue line frames the system control process.

Hi-Tec HS-5086WP digital servomotors were used to generate the roll and pitch movements of the active mirror. These motors have a rotation speed of $60^{o}/s$ according to their data sheet, which implies a rotation speed of $120^{o}/s$ of the virtual camera. That is, system takes 1.5s to change the gaze direction $180^{o}$, which is a reasonable time for underwater systems. Control electronics were encapsulated in a stainless steel cylinder of its own design. Necessary ports were added to communicate with the servos and with the robot by means of optical fiber (only port available in the platform). A photo of the prototype is shown in Figure 11. To perform visual tracking experiments, the AUV was initialized in hovering mode. We assume a static robot and a moving target (red ball) which is not initially in the field of vision. A multidirectional scanning routine moves the active mirror initially over the entire span of the virtual camera in search of the red ball. Once the object is found, the scanning routine is switched to the visual tracking control strategy described in the Section 3.5. When the target is lost, previous information about the centroid of the object is used, however, if no information is available, the system uses the last prediction for the next 30 frames to search for the target. If the object is still not found, the environment is re-scanned to search for it. The workflow of the algorithm is shown in Figure 12. Several tests were carried out at 10–12 m of depth. In all experiments, a skill diver guided the target while swimming along the periphery of the AUV, making trajectories in an open spiral path starting at the same depth of the robot and then ascending and moving away. Supplemental material to this article includes three of the experiments, showing a recording of our AUV’s vision system in the field trials. The download links are referenced in the Supplementary Material section.

During the execution of the experiments, even though the object to track were lost at some time intervals due to the lighting variations, the active system was able to find it and continue to track it. Error plots and Supplementary Videos show these instants, which are particularly present when the ball is on top of the robot and the view direction points toward the sea surface (sunlight). It is important to mention that in all experiments, the object of interest is being detected despite the high brightness in the scene produced by the Sun, thus resulting in successful experiments despite poor lighting conditions and visibility. A clear limitation of our evaluation framework is not being able to give numerical indicators, since that would require precise knowledge of the trajectory followed of the object with respect to the robot’s position and actuators of our mirror-based active system.

Supplementary Video S1 shows all the stages involved in detection and tracking of the target. First, the image obtained by the robot camera is shown, where the different areas of mirrors $M_{1}$ and $M_{2}$ can be observed. Then the segmentation of the area corresponding to $M_{2}$ and its projection using homography matrix are presented. Subsequently, the result of color segmentation in the HSV space is displayed, where the red colors are highlighted. At the end of the video the object tracking process is presented.

**FIGURE 13:**
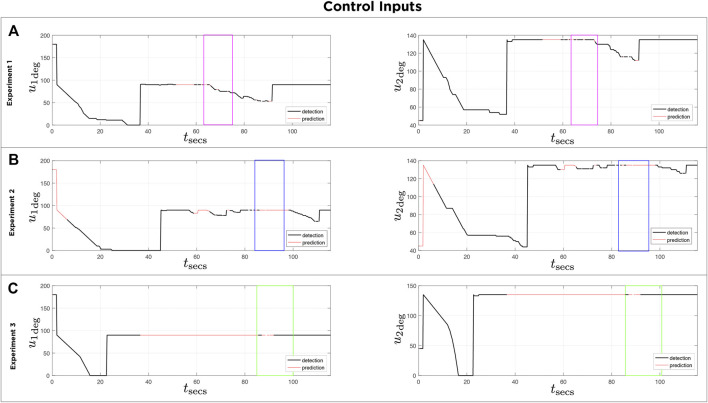
For each of the three experiments **(A**,**B**,**C)**: Reference signals $u_{1}$ and $u_{2}$ calculated using proposed control law. Black lines indicate time intervals when target was detected. Red lines indicate time intervals when controller used the Kalman filter prediction.

**FIGURE 14:**
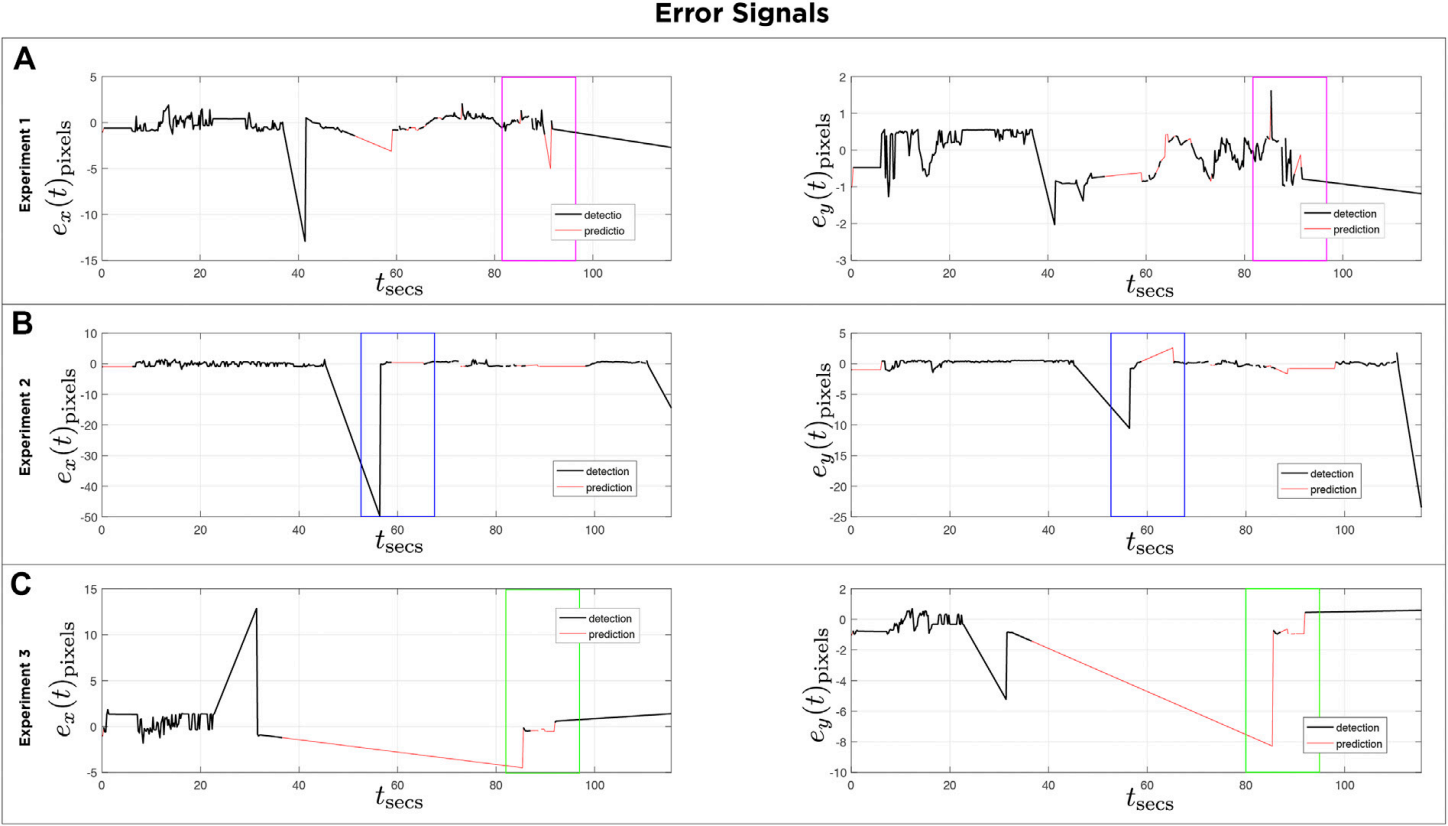
For each of the three experiments **(A**,**B**,**C)**: Error plots signals e(x) and e(y) used for control law in three experiments. Black lines indicate time intervals when target was detected. Red lines indicate time intervals when controller used the Kalman filter prediction.

Figure 13 shows the error signals defined in pixels on both axis of the homography image over time for three different experiments, and the corresponding control signals of the servomotors are depicted in Figure 14. The black lines denote the part of the execution where the detector finds an object in the scene. The red lines show the prediction made with the Kalman filter. Note that in Figures 13A, 14A, the purple boxes indicate the time intervals when the detector loses sight of the moving object. However, by using the predictions of the Kalman filter the tracking of the object is recovered (see Supplementary Video S2). In Figures 13B, 14B, the mirror only moves following the prediction information (see Supplementary Video S3). Figures 13C, 14C illustrate circle (ball) detections, however due to poor lighting the tracking becomes impossible (see Supplementary Video S4). The first 30 s of each experiment corresponds to a preliminary routine that verifies functional communication.

Supplementary Videos S2, S3, S4 show the performance of the system in the field tests. However, by tuning some parameters of the detection algorithm (color threshold and width-height ratio) in offline tests, the detection results improved significantly. Supplementary Video S5 shows a comparison between detection with the original parameters used in the field test and offline detection using different parameters (a greater detection range in the H channel).

## 5 Conclusion and Future Work

The optic mirror-based system presented has the ability to change the direction of view 360° around the AUV and it can have azimuth elevations to detect objects above the robot in order to perform more complex evasive maneuvers.

Fine-tuning of the controller’s gains will allow rapid change of gaze direction, as the angle of view ratio changes twice as much as the movement of the mirror (reflection properties).

Despite the variation of the hydrodynamic parameters of the system, with a suitable ballasting, the AUV can achieve neutral buoyancy.

The method based on HSV color detection is a functional alternative despite poor lighting conditions, also, it has low computational cost and can be implemented for real-time navigation. The right tuning of the tracker’s parameters for the desired color selection will improve its performance. However, the detection process can be modified according to the task, such as the detection of marine fauna, coral species, free space to navigate, divers, rocks, etc., and easily integrated with the proposed controller (PD), considering that the design of the mirror system makes easy the implementation of simple control laws.

Future work includes path planning strategies using the vision system in closed loop with the robot, to be applied to navigation and exploration schemes already studied. As for the prototype itself, it needs an activation and deactivation mechanism, to be able to integrate it into the robot’s cameras and use its maximum resolution. Another point to improve is the automatic calibration of the mirror system. Since the servomotors were used to make this prototype, there is no direct feedback on the state of the system. In addition, a visual estimate is subject to camera calibration and is still difficult to achieve since for small variations in the system there are small image modifications when the normal to the gaze-mirror $M_{1}$ approaches the axis of the main view projected by $M_{2}$.

Another important point is the robot’s communication protocol, as one of the design premises was not to make irreversible modifications to the AUV. In the proposed prototype the only available communication port was used: optical fiber Ethernet. In case the AUV had an additional USB port that could be used for data transmission and even activation, the design of the mirror system would be drastically improved, making it more compact, simpler and increasing the autonomy time.

## Data Availability

The datasets generated for this study are available on request to the corresponding author.
